# Supplementary material for: Stage-Specific Toxicity of Novaluron to Second-Instar Spodoptera frugiperda and Plutella xylostella and Associated Enzyme Responses
Source: Insects. 2025 Oct 15;16(10):1051. doi: 10.3390/insects16101051 (PMC12565172; doi:10.3390/insects16101051)
Supplement: Supplementary file 1 [file insects-16-01051-s001.zip › insects-3890904-supplementary.pdf]

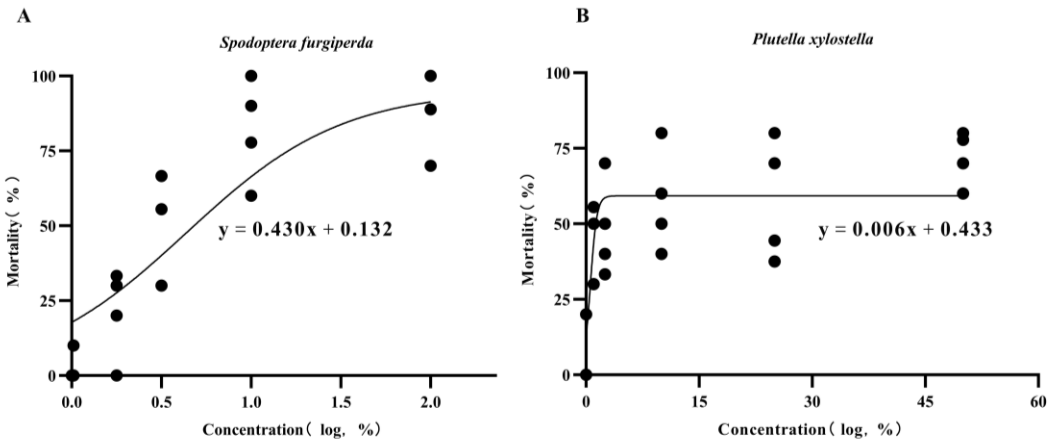

Figure S1. Dose–response relationships of the two species

Figures A and B show the dose–response relationships for the two species. Each point represents the mean of replicates with 95 % confidence intervals. The curve is fitted by a probit/logit model with 95 % confidence interval band.

Table S1. Model details for dose–response analysis of *Spodoptera frugiperda* and *Plutella xylostella*

Species	Link Function	Slope ± SE	Intercept ± SE	Heterogeneity/χ²	AIC/Deviance	Number of Larvae per Dose	Control Mortality (%)	Abbott Correction Applied
Spodoptera frugiperda	y=0.430x+0.132	1.8256 ± 0.309	-3.5451 ± 0.567	0.25 (p=0.617)	24.69	10	0	No
Plutella xylostella	y=0.006x+0.433	2.1356 ± 0.345	-4.2345 ± 0.678	0.35 (p=0.553)	22.45	10	0	No

Table S2. Raw mortality data for *Spodoptera frugiperda* across different concentrations

Concentration (mg/L)	Deaths	Total	Mortality Rate (%)
0	0	10	0.00
0	0	10	0.00
0	0	10	0.00
0	0	10	0.00
0.01	0	10	0.00
0.01	0	9	0.00
0.01	0	10	0.00
0.01	1	10	10.00
0.25	3	10	30.00
0.25	3	9	33.33
0.25	2	10	20.00
0.25	0	10	0.00
0.50	5	9	55.56
0.50	3	10	30.00
0.50	6	9	66.67
0.50	6	9	66.67
1.00	9	9	100.00
1.00	7	9	77.78
1.00	6	10	60.00
1.00	9	10	90.00
2.00	10	10	100.00
2.00	7	10	70.00
2.00	8	9	88.89
2.00	7	10	70.00

Table S3. Replicate-level mortality data for *Spodoptera frugiperda*

Concentration (mg/L)	Replicate	Deaths	Total
0	1	0	10
0	2	0	10
0	3	0	10
0	4	0	10
0.01	1	0	10
0.01	2	0	9
0.01	3	0	10
0.01	4	1	10
0.25	1	3	10
0.25	2	3	9
0.25	3	2	10
0.25	4	0	10
0.50	1	5	9
0.50	2	3	10
0.50	3	6	9
0.50	4	6	9
1.00	1	9	9
1.00	2	7	9
1.00	3	6	10
1.00	4	9	10
2.00	1	10	10
2.00	2	7	10
2.00	3	8	9
2.00	4	7	10

Table S4. Raw mortality data for *Plutella xylostella* across different concentrations

Concentration (mg/L)	Deaths	Total	Mortality Rate (%)
0	2	10	20.00
0	0	10	0.00
0	0	10	0.00
0	0	10	0.00
1.00	5	10	50.00
1.00	3	10	30.00
1.00	5	9	55.56
1.00	3	10	30.00
2.50	4	10	40.00
2.50	5	10	50.00
2.50	3	9	33.33
2.50	7	10	70.00
10.00	5	10	50.00
10.00	6	10	60.00
10.00	4	10	40.00
10.00	8	10	80.00
25.00	4	9	44.44
25.00	3	8	37.50
25.00	7	10	70.00
25.00	8	10	80.00
50.00	7	9	77.78
50.00	7	10	70.00
50.00	8	10	80.00
50.00	6	10	60.00

Table S5. Replicate-level mortality data for *Plutella xylostella*

Concentration (mg/L)	Replicate	Deaths	Total
0	1	2	10
0	2	0	10
0	3	0	10
0	4	0	10
1.00	1	5	10
1.00	2	3	10
1.00	3	5	9
1.00	4	3	10
2.50	1	4	10
2.50	2	5	10
2.50	3	3	9
2.50	4	7	10
10.00	1	5	10
10.00	2	6	10
10.00	3	4	10
10.00	4	8	10
25.00	1	4	9
25.00	2	3	8
25.00	3	7	10
25.00	4	8	10
50.00	1	7	9
50.00	2	7	10
50.00	3	8	10
50.00	4	6	10

Supplementary Information: Detailed Methods for Determination of Detoxifying Enzyme Activities and Ecdysteroid Content

Determination of Detoxifying Enzyme Activities

1. **Enzyme Source Preparation**: The selected samples were ground in liquid nitrogen and mixed with PBS buffer solution at a ratio of 1:9 (g/ml). The mixture was centrifuged at 5000 r/min at 4 °C for 10 minutes, and the supernatant was collected for use.
2. **Standard Sample Dilution**: The standards were diluted to different concentration gradients according to the kit instructions.
3. **Incubation After Sample Addition**: The microplate was set up with blank, standard, and sample wells. A total of 50 μL of the diluted sample (5-fold) was added to the sample wells, and 50 μL of the standard sample was added to the standard wells. The plate was gently mixed, sealed with a cover film, and incubated at 37 °C for 30 minutes.
4. **Washing**: The liquid in the microplate was discarded, and each well was filled with a 20-fold diluted washing solution. After standing for 30 seconds, the liquid was discarded, and the plate was patted dry. This washing process was repeated 5 times.
5. **Enzyme Addition and Incubation**: A total of 50 μL of enzyme-labeled reagent was added to each well (except the blank well) and incubated at 37 °C for 30 minutes.
6. **Second Washing**: The same washing method was used.
7. **Color Development**: A total of 50 μL of color developer A was added to each well, followed by 50 μL of color developer B. The plate was gently mixed and incubated at 37 °C in the dark for 10 minutes.
8. **Termination**: A total of 50 μL of stop solution was added to each well to terminate the reaction.
9. **Measurement**: The absorbance (OD value) of each well was measured at 450 nm with the blank well set to zero. A standard curve was plotted on graph paper, and the concentration of the test samples corresponding to the OD values was

determined from the standard curve. The actual concentration of the samples was obtained by multiplying the concentration values by the dilution factor.

Determination of Ecdysteroid Content

1. **Enzyme Source Preparation**: The selected samples were ground in liquid nitrogen and mixed with PBS buffer solution at a ratio of 1:9 (g/ml). The mixture was centrifuged at 3000 r/min at 4℃ for 15 minutes, and the supernatant was collected for use.
2. **Setting Up Standard and Sample Wells**: A total of 50 μL of different concentrations of standards was added to the standard wells.
3. **Sample Well Treatment**: A total of 10 μL of the test sample was added to the sample wells, followed by 40 μL of sample dilution solution; no addition was made to the blank wells.
4. **Enzyme Addition and Incubation**: A total of 100 μL of horseradish peroxidase (HRP)-labeled detection antibody was added to each well (except the blank well). The plate was sealed with a cover film and incubated at 37 ℃ for 60 minutes in a water bath or incubator.
5. **Washing**: The liquid was discarded, and the plate was patted dry on absorbent paper. Each well was filled with washing solution, stood for 1 minute, and then the liquid was discarded and patted dry on absorbent paper. This washing process was repeated 5 times.
6. **Color Development**: A total of 50 μL of substrate A and 50 μL of substrate B were added to each well, and the plate was incubated at 37 ℃ in the dark for 1.
